# Supplementary material for: Genetic Determinants of Acinetobacter baumannii Serum-Associated Adaptive Efflux-Mediated Antibiotic Resistance
Source: Antibiotics (Basel). 2023 Jul 11;12(7):1173. doi: 10.3390/antibiotics12071173 (PMC10376123; doi:10.3390/antibiotics12071173)
Supplement: Supplementary file 1 [file antibiotics-12-01173-s001.zip › antibiotics-2470772-supplementary.pdf]

Supplementary Materials

Supplemental Figure S1.

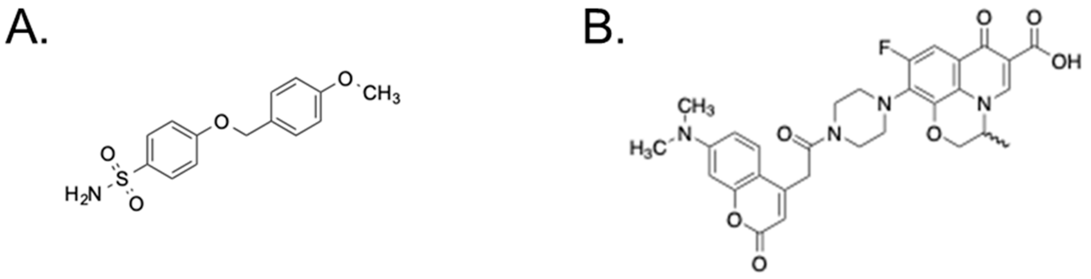

Supplemental Figure S1. Chemical structures. Shown are the structures of DMACA-Olfoxacin (Panel A) and AE0003 (Panel B).

Supplemental Figure S2.

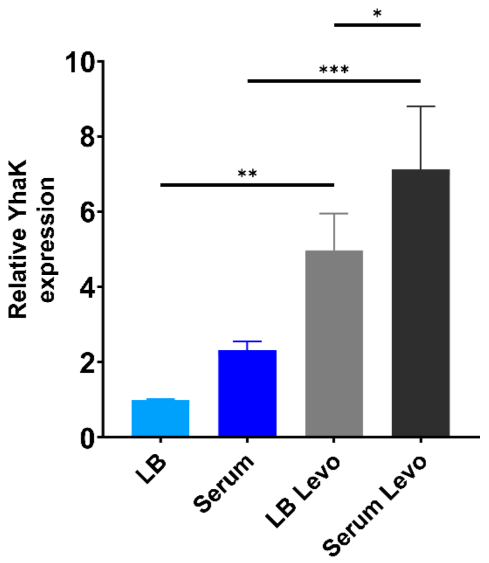

Supplemental Figure S2. Quantitative real time PCR measures of YhaK transcription. Shown are the relative expression levels of *yhaK* within *A. baumannii* strain 98-37-09 during growth in LB or human serum ± 0.2 μg ml^{-1} levofloxacin. All data normalized to LB grown

cells in the absence of antibiotic. Standard deviation shown; statistical difference (One-way ANOVA, * P ≤ 0.05, ** P ≤0.01 and *** P ≤0.001).

Supplemental Figure S3

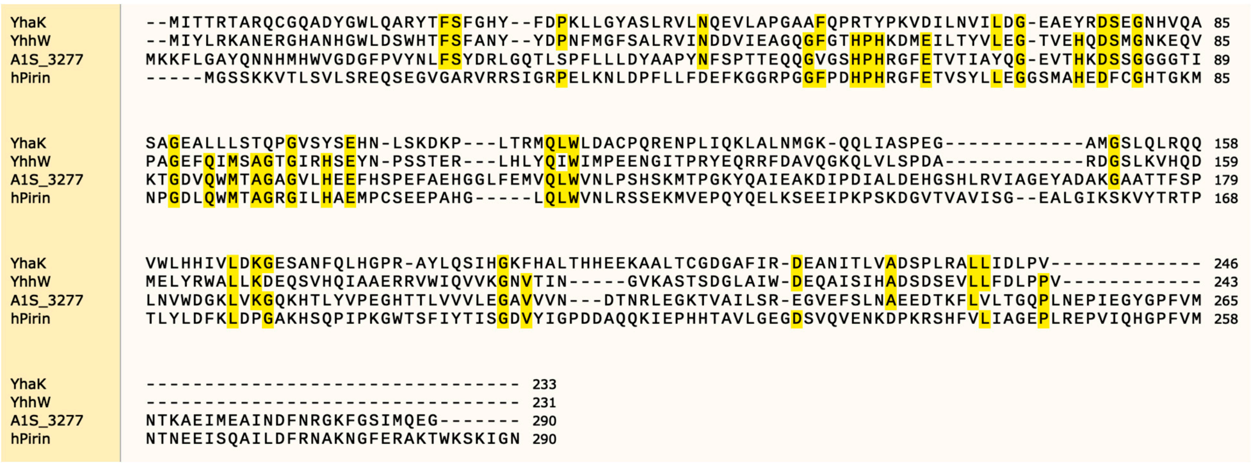

Supplemental Figure S3. ClustalW alignment of *Escherichia coli* pirin-like protein YhaK, *E. coli* 2,3 dioxygenase YhhW, human pirin protein (hPirin), and *A. baumannii* YhaK pirin-like protein (A1S_3277). Matching amino acid residues are highlighted in yellow, while the location of four conserved metal binding residues (His57, His59, His101, and Glu103) are highlighted by red boxes and corresponding notation. Amino acid positions are from *E. coli* YhhW.
